# Morphodynamics and management challenges for beaches in modified estuaries and bays

**DOI:** 10.1017/cft.2024.7

**Published:** 2024-08-27

**Authors:** Ana Vila-Concejo, Thomas E. Fellowes, Shari Gallop, Irene Alejo, Donatus B. Angnuureng, Javier Benavente, Jorn W. Bosma, Emmanuel K. Brempong, Pushpa Dissanayake, Md Yousuf Gazi, Rita González-Villanueva, Ricardo Guimarães, David M. Kennedy, John L. Largier, Marlies A. van der Lugt, Juan Montes, Mara Orescanin, Charitha B. Pattiaratchi, Luci Cajueiro Carneiro Pereira, Remo Luan Marinho da Costa Pereira, Timothy Price, Maryam Rahbani, Laura del Río, Michael Rosenthal, Matthieu de Schipper, Anne M. Ton, Lukas WinklerPrins, Zhongyuan Chen

**Affiliations:** 1 Geocoastal Research Group, School of Geosciences and Marine Studies Institute, Faculty of Science, The University of Sydney, and Sydney Institute of Marine Science, Chowder Bay, Sydney, NSW, Australia; 2 School of Science, University of Waikato, Tauranga, New Zealand; 3 Environmental Research Institute, University of Waikato, Hamilton, New Zealand; 4 Marine Research Centre (CIM), University of Vigo, Vigo, Spain; 5 Centre for Coastal Management—Africa Centre of Excellence in Coastal Resilience (ACECoR), University of Cape Coast, Cape Coast, Ghana; 6 INMAR/Earth Sciences Department, University of Cádiz, Cádiz, Spain; 7 Faculty of Geosciences, Department of Physical Geography, Utrecht University, Utrecht, The Netherlands; 8 Department of Fisheries and Aquatic Sciences (DFAS), University of Cape Coast, Cape Coast, Ghana; 9 LEGOS, OMP, UMR 5566 (CNES-CNRS-IRD-University of Toulouse), Toulouse, France; 10 Coastal Geology and Sedimentology, Institute of Geosciences, University of Kiel, Germany, and NLWKN, Lower Saxony Government, Germany; 11 Department of Geology, University of Dhaka, Dhaka, Bangladesh; 12 Programa de Engenharia Oceânica, Universidade Federal do Rio de Janeiro, Rio de Janeiro, Brazil; 13 School of Geography, Earth and Atmospheric Sciences, The University of Melbourne, Parkville, VIC, Australia; 14 Coastal and Marine Sciences Institute, University of California Davis, Bodega Bay, CA, USA; 15 Department of Environmental Science & Policy, University of California Davis, Davis, CA, USA; 16 Deltares, Delft, The Netherlands; 17 Civil Engineering and Geosciences, Delft University of Technology, Delft, The Netherlands; 18 Department of Oceanography, Naval Postgraduate School, Monterey, CA, USA; 19 Oceans Graduate School & The UWA Oceans Institute, The University of Western Australia, Perth, WA, Australia; 20 Universidade Federal do Pará Instituto de Estudos Costeiro, Bragança, Brazil; 21 Universitat Politècnica de Catalunya - BarcelonaTech, Barcelona, Spain; 22 Department of Marine Science and Technology, University of Hormozgan, Bandar Abbas, Iran; 23 Rhelm Consulting, Neutral Bay, NSW, Australia; 24 Civil & Environmental Engineering, University of California Berkeley, Berkeley, CA, USA; 25 State Key Laboratory for Estuarine and Coastal Research, East China Normal University, Shanghai, China

**Keywords:** estuarine beaches, coastal evolution, ecosystem services, global coasts

## Abstract

There is a relative lack of research, targeted models and tools to manage beaches in estuaries and bays (BEBs). Many estuaries and bays have been highly modified and urbanised, for example port developments and coastal revetments. This paper outlines the complications and opportunities for conserving and managing BEBs in modified estuaries. To do this, we focus on eight diverse case studies from North and South America, Asia, Europe, Africa and Australia combined with the broader global literature. Our key findings are as follows: (1) BEBs are diverse and exist under a great variety of tide and wave conditions that differentiate them from open-coast beaches; (2) BEBs often lack statutory protection and many have already been sacrificed to development; (3) BEBs lack specific management tools and are often managed using tools developed for open-coast beaches; and (4) BEBs have the potential to become important in “nature-based” management solutions. We set the future research agenda for BEBs, which should include broadening research to include greater diversity of BEBs than in the past, standardising monitoring techniques, including the development of global databases using citizen science and developing specific management tools for BEBs. We must recognise BEBs as unique coastal features and develop the required fundamental knowledge and tools to effectively manage them, so they can continue providing their unique ecosystem services.

## Graphical abstract

**Figure:**
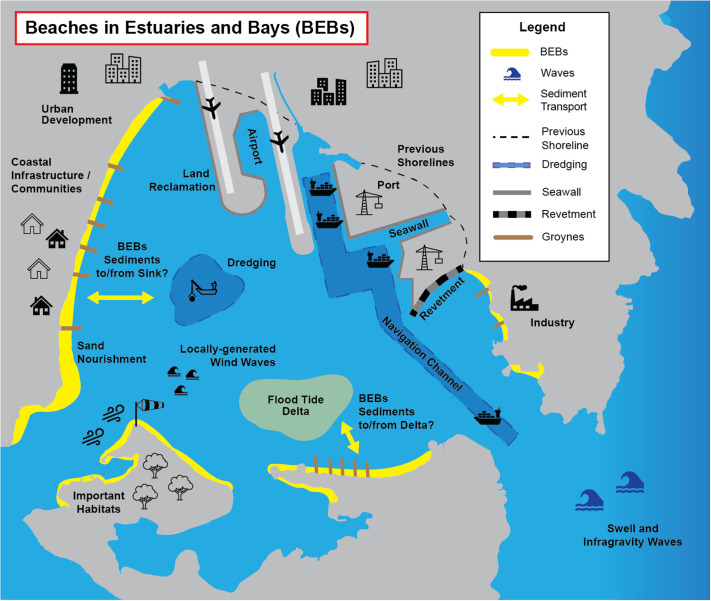

## Impact statement

We bring together an international team of researchers to bring a comprehensive review and perspective on beaches on estuaries and bays (BEBs). Our work delves into recent research drawn from eight case studies spanning the Africa, Americas, Asia, Australia and Europe. By contextualising this research within the existing literature on BEBs, we have achieved a unique perspective that sheds light on the intricate challenges and complexities involved in conserving and managing these delicate ecosystems. We believe this perspective offers valuable insights into the field. Furthermore, our paper outlines our vision for the trajectory of future research in this domain. We delineate a series of progressive steps that should serve as guideposts for upcoming research on BEBs, aiming to facilitate a more holistic understanding of these environments. Our findings show that the key to setting the future research agenda for BEBs is to first broaden our research focus to include a greater diversity of BEBs, based on the great variation in the relative importance of the many factors that drive BEB morphodynamics. We recommend including more focus on mapping and monitoring BEB locations and morphology and long-term monitoring of hydrodynamic processes. Future studies should consider BEB evolution in relation to evolution and processes of the whole the estuary/bay to identify potential mitigation measures based on nature-based solutions.

## Introduction

When considering beaches in estuaries and bays (BEBs), generally low energy, narrow landforms come to mind. However, the environmental settings and morphology of such beaches are highly diverse in terms of planform, cross-shore profile shape and hydrodynamic drivers. BEBs can be exposed to various combinations of ocean-generated waves and those generated inside the estuary/bay, in addition to other hydrodynamic forcing such as currents generated by rivers and tides (Vila-Concejo et al., 2020). While geological inheritance is a first-order control on the location, shape, volume and stability of BEBs, the geology can also control the contemporary dynamics, for example pocket BEBs between rocky outcrops (Gallop et al., 2020a). Moreover, many BEBs are in highly modified estuaries and bays, with hard engineering works and dredging also being important controls on their form and behaviour (Fellowes et al., 2021). Indeed, engineering interventions in estuaries and bays such as port development have caused the loss of entire BEBs systems, or their creation through artificial means (e.g. nourishments associated with groynes).

**Figure 1.**
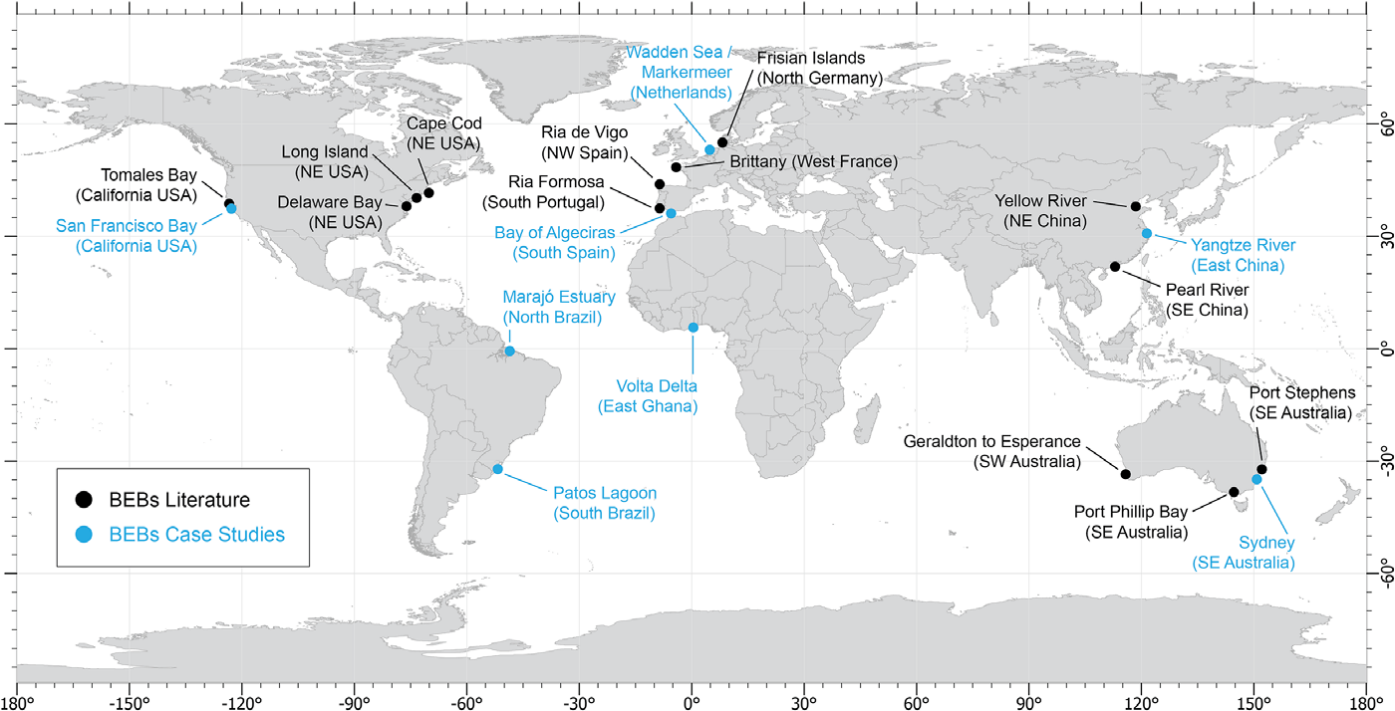
World map of BEBs in the peer-reviewed literature (black dots) and case studies presented in this paper (blue dots).

There is a relative lack of research, models and management tools for BEBs compared with open ocean beaches (Figure 1) (e.g. Vila-Concejo et al., 2020; Ton et al., 2021). Based on observations in the NE USA, Nordstrom (1992) provided a general background to BEBs, which was followed by other work on low-energy and sheltered beaches, such as Hegge et al. (1996) on reef-controlled, sheltered beaches on the open coast and Jackson et al. (2002), who focused on non-estuarine BEBs. There have been several classifications developed for low-energy beaches, but not specifically for BEBs. This includes the Short (2006) and Short and Woodroffe (2009) classifications of tide-modified/dominated beaches focused on the open coast; the work of Travers (2007), Travers et al. (2010) on the morphodynamics of BEBs in SW Australia and classifications of fetch-limited beaches based on the importance of wave, tidal and river forcing (Freire et al., 2013, 2009). Importantly, in all these studies locally generated wind waves, sometimes modulated by the tidal forces, were considered the major control for BEBs morphodynamics. There have also been studies on the dynamics of specific BEBs in Spain (Alejo et al., 2005; Costas et al., 2005; Gonzalez-Villanueva et al., 2007; Bernabeu et al., 2012), Portugal (Carrasco et al., 2012, 2008; Freire et al., 2013), France (Dissanayake et al., 2021), Germany (Dissanayake and Brown, 2022), Hong Kong, China (Yu et al., 2013), SE Australia (Kennedy, 2002; Gallop et al., 2020b; Fellowes et al., 2021; Rahbani et al., 2022) and California, USA (Winkler-Prins et al., 2023) (Figure 1).

While many BEBs have been lost to urbanisation, the remaining BEBs in urban environments provide important places for people to connect with nature and ecosystem services such as providing habitat and feeding areas and protective buffers for wetlands (Nordstrom and Jackson, 2012), as well as providing safe swimming areas (Largier and Taggart, 2006). This socio-ecological role is highlighted by artificial BEBs created to upgrade flood defences and to provide a more natural transition between land and water than traditional shore protection works, such as in the Netherlands (Ton et al., 2023) and California (SFEI and Baye, 2020). While many BEBs are often protected from large waves, severe erosion can still occur when storms come from directions that can propagate large swells inside estuaries/bays (Gallop et al., 2020b) In fact, further research has shown that BEBs in those environments are mostly controlled by the swell energy propagating into the estuaries and bays (Rahbani et al., 2022). Moreover, recovery of BEBs after erosive events can be slow and take years (Nordstrom, 1980; Costas et al., 2005; Fellowes et al., 2021). As such, with their generally low-lying nature, sensitivity to changes in wave direction or extreme winds and slow recovery, BEBs are highly sensitive to climate-driven changes in wave forcing and impacts of compound events including precipitation and storm surge. Maintaining healthy BEBs contributes to the United Nations Sustainable Development Goals number 11, 14 and 15 (UN, 2015). Vila-Concejo et al. (2020) provide a complete overview on the geological setting and oceanographic conditions that determine where BEBs form and what they look like.

Despite recent increased research on BEBs, the need remains to better understand their processes to develop models to underpin their management. We take a step towards this here by bringing together an international group of BEB researchers and practitioners to share and consolidate understanding of BEB morphodynamics and set a collective research agenda. Our aim is to highlight diverse morphologies of BEBs in estuaries, bays and coastal lagoons from around the world and their management issues. This paper provides case studies from seven regions with BEBs in Ghana, Brazil, USA, Australia, China, Spain and the Netherlands, selected for their diverse morphodynamics and management issues. The case studies include pristine BEBs (e.g. Northern Brazil) and with large anthropogenic impact and undergoing erosion (e.g. China). The tides in the cases studied go from microtidal (e.g. SE Australia) to mesotidal (e.g. San Francisco, USA) to macrotidal (Northern Brazil), and they include BEBs that never receive any swell energy (e.g. the Netherlands) and those that may be controlled by swell (e.g. SE Australia). This is followed by discussion of the key challenges in conserving and managing BEBs in modified estuaries, bays and lagoons, and our perspectives on the future agenda of BEB research against the backdrop of climate change and increased infrastructure development resulting from population growth.

## Case studies

### BEBs in the lower Volta Delta (Ghana, West Africa)

West African beaches have undergone rapid changes in recent years due to natural and anthropogenic factors (Alves et al., 2020). The Volta Delta situated on Ghana’s eastern coast is a prime example of a highly dynamic and erosion-prone region (Figure 1). The Volta Estuary of the Volta Delta is at the mouth of three major West African rivers that drain large parts of Ghana, Togo, Burkina Faso and smaller portions of Côte d’Ivoire, Mali and Benin and accommodate many BEBs that have great significance. These BEBs typically front narrow sandy barriers that are facing significant erosion, posing risks to coastal settlements and natural ecosystems. On the open coast, beaches are wave-dominated, with an average H_s_ of 1.4 m and peak wave period (T_p_) of 11 s (Angnuureng et al., 2020). The tidal range is about 1 m (Addo et al., 2018). The Volta Delta coast has extensive swamps with intermittent mangrove areas of predominantly red mangrove (Kortatsi et al., 2005) and savannah woodlands (Boatema et al., 2013). Due to increased flooding and the construction of the Akosombo Dam in 1963 on the Volta River, the BEBs inside the Volta Estuary and adjacent open-coast beaches have experienced rapid shoreline transgression. For example, the open-coast Fuveme community, west of the mouth, lost 37% of its area, resulting in the displacement of people and the destruction of houses with the entire community being lost in November 2021. Ada Foah beach to the east of the mouth suffered from both erosion and flooding, also causing the loss of schools and settlements (Addo et al., 2018). Wave overtopping occurs on the coastal area of the Delta due to its low-lying nature, thus causing salinisation within the BEBs. This has the potential to degrade the freshwater ecosystems within the Delta, perhaps an unexpected consequence arising from BEB erosion. Although there is a lack of studies on the evolution of BEBs in this estuary, it is evident that like the beaches on the open coast, most of the major BEBs near the estuary entrance have also undergone severe erosion over decades since the dam construction. As the shoreline has adjusted to changes in catchment sediment yields, beach erosion has been further exacerbated as residents have attempted ad hoc hard infrastructure protection such as placing rocks on the beach. To effectively manage the BEBs in the Volta Estuary, there is a need for a deeper understanding of their processes, targeted models and management practices with particular attention being paid to the regional and local sediment budgets.

### BEBs in south and Southeast China (Asia)

BEBs in China, often encompassing tidal flats, are extensively developed along the S and SE coasts and associated to large rivers like the Yellow, the Yangtze and the Pearl (Figure 1). The most prominent geographical setting of these BEBs is the high supply of fluvial materials (sediment, discharge and nutrients), combined with strong coastal tidal/wave currents and the presence of densely urbanised landscapes (Zhang et al., 2016; Wu et al., 2018). However, the construction of large dams along with rising marine hazards, for example saltwater intrusion and coastal erosion, has largely affected the habitats on BEBs (Chen et al., 2010; Wu et al., 2016). Consequently, dams now prevent the transport of sufficient sediments into the estuaries, and therefore, BEBs are eroding with hard engineering structures in place to prevent coastal erosion. This is particularly concerning when considering potential seasonal high energy conditions induced by tropical storms (typhoons) from the West Pacific Ocean. Examples of these profoundly modified BEBs can be found in the metropolitan city of Shanghai and Guangzhou inhabited by 18–20 million people. These socio-ecological settings in China’s estuaries are alarming to the stakeholders underscoring the urgent need for legislative action at both municipal and national levels to curb further degradation of BEBs.

### BEBs on the Amazon and South Atlantic coasts (Brazil, South America)

**Figure 2.**
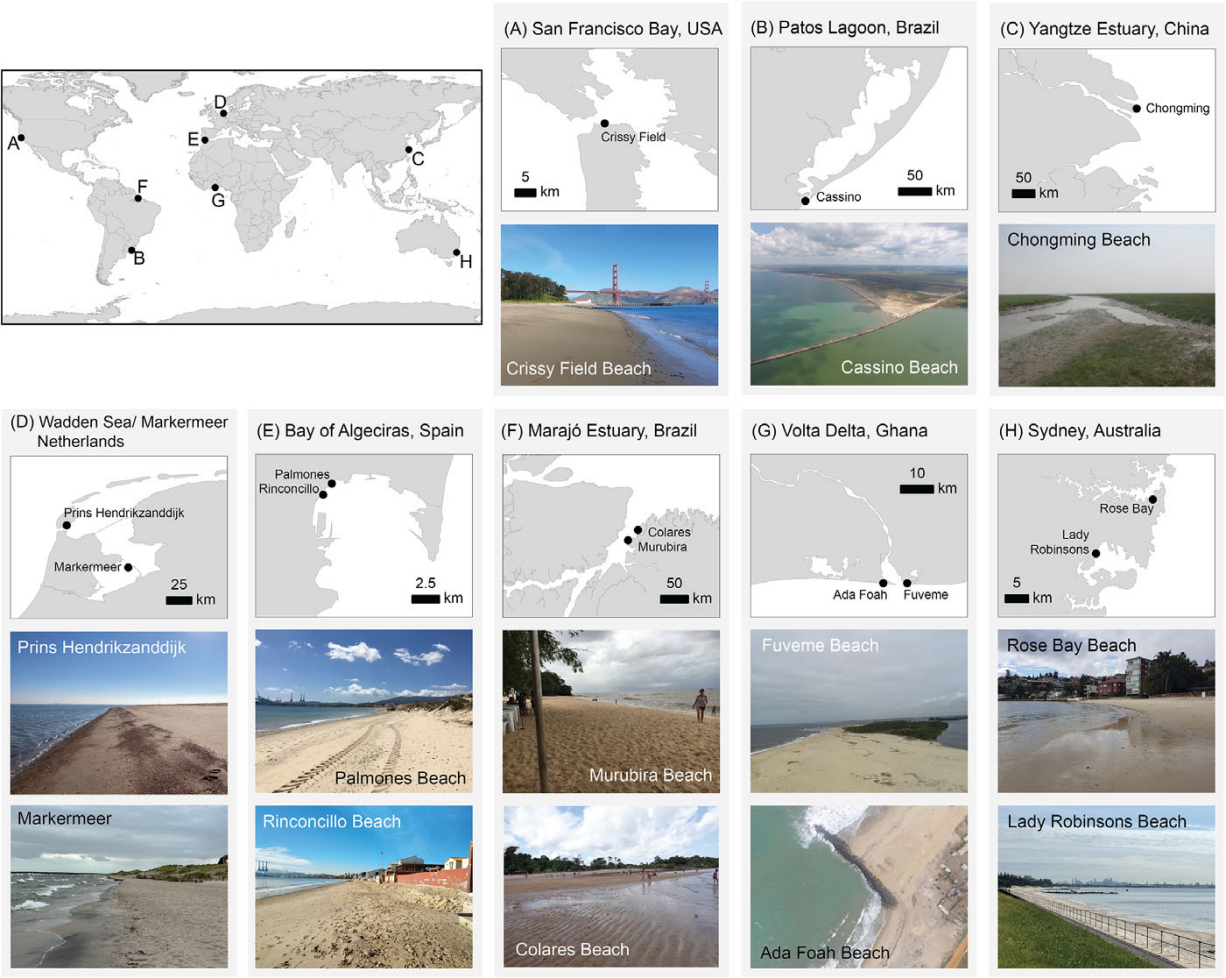
BEBs case study locations and photographs. For world location, please refer to Figure 1.

Brazil has a broad range of BEBs in its diverse estuarine systems. BEBs along the Marajó estuary, part of the Amazon River estuarine system (Figure 1), are exposed to macro-/mesotides (3–6 m) that modulate the low-to-moderate waves (H_s_ = 0.5–1.5 m) propagating over the inter- to subtidal sand-/mudbanks (Pereira et al., 2016). On the eastern side of the estuary, there are 157 beaches along 265 km of mangrove-dominated shoreline, intersected with rivers and creeks forming bays, distributary islands and extensive tidal shoals (Anthony et al., 2010). Some of these BEBs are narrow (up to 50–70 m width) and have a high-gradient intertidal zone (> 5°) with reflective characteristics, composed of medium sand (e.g. Murubira; Figure 2). Other BEBs have intermediate characteristics, are wide (up to 350–450 m) and have a low-gradient intertidal zone (1°) (e.g. Colares; Figure 2).

In southern Brazil, the microtidal (0.25 m tidal range) Patos Lagoon (Figure 1) plays a significant role in the regional sediment dynamics (Marques et al., 2010). Export rates of suspended sediment to the coast are up to 1.37 10^7^ t/year of total suspended matter (Marques et al., 2010). The area of fresh-/salt water mixing extends 60 km from the lagoon’s entrance, which is mostly composed of fine sand in the shallower sections transitioning to silt and clay within the deeper channels (Marques et al., 2010). BEB morphodynamics inside this estuary are controlled largely by the river discharge, together with the wind patterns. For example, Praia do Laranjal (Figure 2), a BEB bounding the west jetty of the Patos Lagoon mouth, is highly dissipative with a low intertidal gradient (2°) and mostly wave-dominated (H_s_ = is up to 0.6 m, compared to the average of 1–1.5 m on the open coast) (Tozzi and Calliari, 2000), typically presenting multiple bar systems (Guedes et al., 2009).

One major issue for BEBs in Brazil, both in the North and in the South and especially with climate change, is the lack of specific tools and models with sufficient local data to help inform management.

### BEBs in San Francisco Bay (USA, North America)

San Francisco Bay (Figure 1) has many BEBs, including the urban Crissy Field beach (Figure 2), located 0.5 to 2 km from the entrance on the southern side of the estuary and near the flood tide delta – a sandy BEB connected to a small marsh, facing towards the NE Pacific. Offshore waves that can propagate into the estuary typically approach from the north-west (NW) with H_s_ between 1 and2 m (peak periods >10s), although it is not unusual for H_s_ to exceed 5 m outside the mouth during storms (with T_p_ approaching 20 s). Ocean waves that reach Crissy Field have refracted and decayed with dominant directions from the north-NW and heights between 0.2 and0.4 m. In addition, strong sea breezes over a fetch of 2–3 km can generate high-frequency waves with similar amplitudes, and infragravity waves also occur at this beach. All waves propagate from the west causing strong eastward littoral transport. Given the BEB’s proximity to the bay entrance, the sand supply to Crissy Field is a combination of tidal and wave-driven transport, with sand originating on nearby beaches seawards of the mouth (Barnard et al., 2013) (Figure 1). The BEB encloses a marsh and small 0.07 km^2^ tidal lagoon that closes intermittently, typically when the offshore wave height exceeds 3.5 m driving strong littoral drift across the lagoon mouth (Battalio et al., 2007; Hanes et al., 2011; Hanes and Erikson, 2013). Under low-wave conditions, the tidal currents driven by the 1–2.25 m tides can scour the inlet channel and maintain the lagoon–bay connection (Battalio et al., 2007). When open, outflow from the lagoon builds a small ebb-tide delta and disrupts longshore transport, accounting for a step in the shoreline with the BEB being narrower east of the inlet.

BEBs beyond the influence of ocean waves in San Francisco Bay are shaped by waves generated in the bay (Talke and Stacey, 2003), with longer period and larger waves incident from directions with longer wind fetch. Wind-generated waves can approach BEBs from multiple directions, resulting in seasonal cycles; for example, Marina Bay beach, further into the estuary, is worked by SW wind waves during winter as well as by refracted NW wind waves during summer (Accordino, 2022). Here, and at other BEBs in the bay, compound events result in morphological change, such as sand overwash fans and beach/marsh erosion. Increasingly BEBs are being included in designs for marsh restoration around the bay (SFEI and Baye, 2020).

### Swell-dominated BEBs in SE Australia

The coast of SE Australia is microtidal with mean tidal ranges of 1.6 m and 1.3 m for spring and neap tides, respectively. It receives swells with H_s_ of 1.6 m and a 10-s peak period (Short and Trenaman, 1992). This moderate wave climate has important repercussions for those BEBs located inside estuaries with wide mouths that allow swell penetration (Vila-Concejo et al., 2010; Gallop et al., 2020b). Indeed, the wave energy controlling BEB morphodynamics in those estuaries is dominated by swell waves under all conditions, particularly under high-energy conditions (Rahbani et al., 2022). The relatively recent urban development of Australian cities and the high wave energy in the open coast have led to engineering developments inside estuaries (Figure 2). For example, Sydney Airport and its commercial port were developed in Gamay estuary (Aboriginal name of Botany Bay) and the engineering works including coastal reclamation, river deviation, revetments, seawalls and dredging led to the erosion of urban BEBs that were deemed sacrificial for the sake of urban development (Fellowes et al., 2021). At the same time, some of Australia’s most expensive real estate in Sydney Harbour is protected by BEBs (Figure 2), and some of the most prominent erosion hotspots correspond to BEBs, for example Jimmy’s Beach in Port Stephens (Vila-Concejo et al., 2020, 2010).

### Modified BEBs in the bay of Algeciras (southern Spain, South-Western Europe)

The Bay of Algeciras (Figure 1) faces south into the Strait of Gibraltar, is microtidal (mean spring tidal range 0.98 m) and sheltered from ocean-generated waves. Waves approach mostly from the SE and have significant wave heights (H_s_) less than 0.1 m, with 1.5 m H_s_ being exceeded several times per year (Montes, 2021). The Rinconcillo–Palmones System (RPS) on the NW side of the bay includes an urban beach (Rinconcillo) and a sandspit (Palmones) (Figure 2). The RPS is adjacent to Bahía de Algeciras Port, one of Europe’s most important ports.

The Algeciras port interrupts the prevailing northward longshore drift and was enlarged significantly in 2000 and 2010, currently extending more than 1.5 km into the sea. This modified the local wave patterns adjacent to seawalls and jetties. BEBs are very sensitive to changes in wave direction (Gallop et al., 2020b), and consequently, the RPS is now rotating counterclockwise because of these changes, except for at the spit end, which is controlled largely by currents at the mouth of the Palmones River. Since 2000, the shoreline has prograded at rates over 4 m/yr. at the southern end of the RPS (next to the port), while the northern area has eroded at a rate of around 1 m/yr (Montes, 2021). In areas behind the narrowing beach, there is more frequent damage to private property and infrastructure during storms. As occurs at other BEBs (e.g. Costas et al., 2005; Harris et al., 2020), beach recovery at RPS, which does not usually reach pre-storm state, requires several months of calm conditions (Montes, 2021).

The RPS has a bimodal longshore drift that transports eroded sand alongshore and into deeper areas offshore (Montes, 2021). Northward sediment transport occurs during modal conditions transporting material towards the Palmones river mouth and ebb-tidal delta. From there, sediment can be lost to deeper areas in the Bay of Algeciras as depths greater than 50 m occur very close to the coast. Southward sediment transport occurs during storms, when material is transported from the north, where an eroded dune system occurs (Figure 2) and deposited adjacent to the port. The modified sediment transport pathways, because of the port construction and later expansion, have caused the southward sediment transport mode to now become prevalent.

### Artificial BEBs in the Netherlands (North-Western Europe)

Dutch estuarine and lake shores are often lined with hard (i.e. asphalt, concrete and stone) flood defences, which require regular reinforcement to withstand current and future marine processes. In recent years, the creation of artificial beaches (e.g. Prins Hendrikzanddijk; Figure 2) in front of hard defences is a paradigm shift from reinforcement of old coastal infrastructure with hard material to nature-based or hybrid solutions (Perk et al., 2019). Despite ample experience in nourishing large volumes of sand at the wave-dominated Dutch open coast (Brand et al., 2022), the understanding of artificial BEBs mainly stems from a “learning by doing” approach, in which continuous monitoring is key to understanding and predicting their development, ultimately enabling safety assessments.

The BEBs in the northern Netherlands are subjected to locally generated wind waves with mean H_s_ of 0.1 to 0.3 m, reaching up to 1.5 m. Longshore currents include relatively strong tidal currents (~0.6 m/s) which are strongly influenced by wind-driven circulation (~0.25 m/s) in the (semi-) enclosed regions (Ton et al., 2023). As the nourishment sediment is often coarser than the native material (to limit erosion), the surface armouring provided by these coarser sediments causes beach response to be mostly event-driven.

The subsequent equilibration of the profile and planform shape by natural forces depends on the orientation and geometry of the beach with respect to the hydrodynamic forcing. Cross-shore profile adjustment often involves a strong retreat and steepening of the beach face, coinciding with the development of a more concave upward profile and a relatively stable platform at water depths around the depth of closure (Hallermeier, 1980, 1978), where the surface waves reach the limit of their erosive action (Ton et al., 2021). In addition, longshore drift further redistributes and sorts the nourished sediment, leading to beach rotation, spit formation (10s of meters per year) and the development of cuspate shorelines (van Kouwen et al., 2023).

## Challenges for conserving and managing BEBs in modified estuaries

The case studies above highlight the diversity of the environmental settings and morphodynamics of BEBs, and the many common (and unique) management issues they face worldwide. While BEBs are common globally, they are still relatively small morphological features that require the right balance of conditions to form including accommodation space, sediment supply and wave conditions to build and then maintain the beach. The case studies highlight the variety of tidal (micro- to macrotidal), wave conditions and hydrodynamic circulation that maintains BEBs. This includes swell-dominated environments such as BEBs in SE Australia (e.g. Vila-Concejo et al., 2010; Rahbani et al., 2022) and BEBs near the entrance of San Francisco Bay (e.g. Hanes and Erikson, 2013), through very low energy environments where the main forcing is the wind-driven waves and circulation (Ton et al., 2021), to BEBs where locally generated wind waves are the main forcing (e.g. Nordstrom, 1992; Nordstrom and Jackson, 2012; Winkler-Prins et al., 2023). The role of boat wakes on BEB morphodynamics has also been acknowledged but not studied in depth (e.g. Parnell and Kofoed-Hansen, 2001; Hughes et al., 2007; Bilkovic et al., 2019).

Historically, BEBs have lacked the “status” necessary to consider protection and have often been sacrificed to development. This is obvious both in the case studies and in the published literature. The sacrificial status of BEBs is exacerbated by their sensitivity to erosion combined with slow recovery. Many BEBs exist in highly modified coastal environments with many competing stakeholders who often benefit from this lack of status (e.g. Nordstrom, 1992; Vila-Concejo et al., 2020), for example private property owners wanting their own beach front often via engineered means (e.g. Alterman and Pellach, 2022; Iveson and Vila-Concejo, 2023), coastal infrastructure altering local waves (e.g. Fellowes et al., 2021; Montes, 2021) and dam construction changing the sediment discharge in coastal estuaries (e.g. Ly, 1980; Addo et al., 2018). However, as urban sprawl and gentrification reshape estuarine cities of the world, some of these often-derelict sacrificial BEBs become valued enough to be protected, such as in Gamay, Sydney (Fellowes et al., 2021) and San Francisco Bay (SFEI and Baye, 2020).

**Figure 3.**
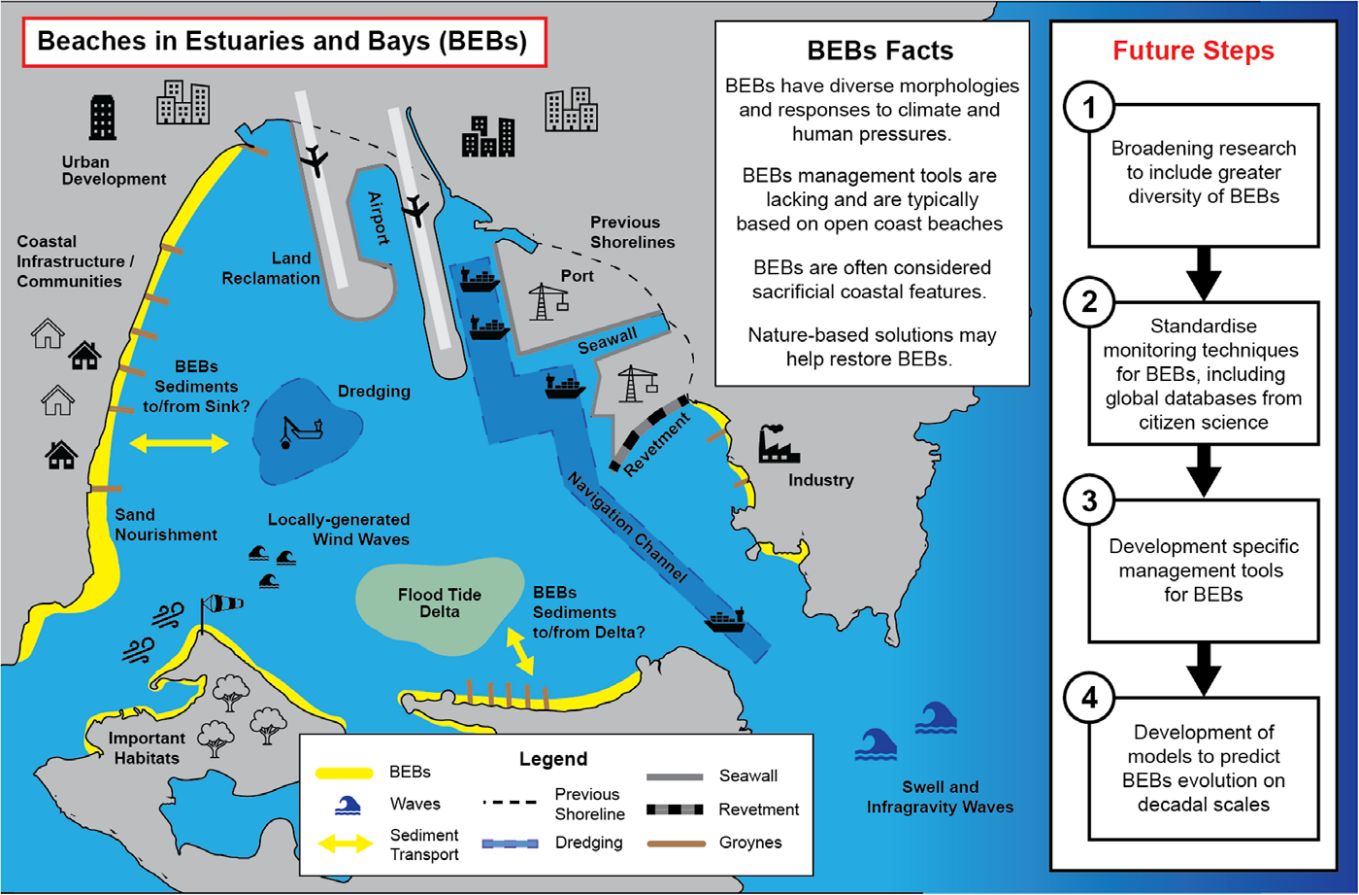
Conceptual diagram showing the challenges of managing BEBs in modified estuaries and a summary of the future steps arising from this paper.

All case studies in this paper emphasise the lack of knowledge, classifications/models and management tools specific for BEBs (Figure 3). The management of open-coast beaches is underpinned by the knowledge on the drivers of erosion and recovery processes on beaches and the existence of classification models. Often, one-size-fits-all management guidelines such as erosion prone area mapping are developed based on open-coast processes which are, in turn, and inappropriately, applied to BEBs. While ocean waves and locally generated wind waves may be the cause of erosion under high-energy events (e.g. Gallop et al., 2020b); in other cases, erosion might be caused by engineering interventions, sometimes nearby, sometimes hundreds of kilometres away, that alter the sediment pathways to the BEBs. Moreover, in the case of some Dutch artificial beaches, it is the wind-driven circulation combined with very-low-energy waves that may cause erosive processes (Ton et al., 2023). Another key consideration is accounting for where eroded sediment goes after being removed (such as during a storm). BEBs seldom have subtidal bars where the eroded sand can be stored; indeed, in the case studies, this is only described for some of the BEBs in Brazil. More often, the eroded sand is transported into the estuary where it can be lost to deep basins/channels or transported to shoals and/or the flood tide delta. In any case, the pathways and mechanisms by which this lost sediment may be restored to the BEBs are unknown or non-existent. The complexity of BEB morphodynamics is exacerbated further in that many have a mixed sediment composition including sand, clay, shells and often gravel (Nordstrom, 1992).

Recent decades have seen the advent of “nature-based” engineering solutions for coastal protection that aim to replicate nature rather than to work against it. Our paper highlights the potential of BEBs for this approach, through their ability to protect crucial human infrastructure, in case of the Dutch artificial beaches, and through their inclusion in marsh restoration projects in the San Francisco Bay. Indeed, BEB research and management constitutes an important example of a socio-ecological challenge where the complexity of the relationships between the ecological and social realms remains unexplained (Diedrich and Tintoré, 2012). Research has shown that comparing what people perceive with what is occurring in environmental management scenarios can help identify potential discords and, hence, shape environmentally significant behaviour (Diedrich and Tintoré, 2012). One example of this complex socio-ecological challenge is the common disagreement between the priorities of beach managers and the needs identified through research. For example, in countries where tourism represents an important industry, management typically prioritises the socio-economic objectives (tourism) over the ecological objectives (e.g. environmental conservation) (Ariza et al., 2008). Despite both academic circles and governance having adopted a holistic view of coastal management including both social and ecological realms, at a lower than national level, private interests and sectorial approaches make the social override of the ecosystem approach (Ariza et al., 2016). Recent developments of nature-based solutions (Temmerman et al., 2013; Narayan et al., 2016) represent opportunities to consolidate a socio-ecological approach to engage with oyster reef restoration, living shorelines and other ecosystem restoration projects in which BEBs should be considered.

## Future steps in BEBs research

In this section, we present four steps to guide future research on BEBs based on our discussions above (Figure 3). The first two are focused on data acquisition, and the last two are focused on tool development.

The key to setting the future research agenda for BEBs is to first broaden our research focus to include a greater diversity of BEBs, based on the great variation in the relative importance of the many factors that drive BEB morphodynamics. For example, including different types of estuaries and bays in different parts of the world as current research is clustered sporadically around the globe (Figure 1) and tends to focus mostly on wind waves as the key driver, with little focus on swell, infragravity and tidal waves. Moreover, the influence of anthropogenic activity such as reclamation and impacts from boat wakes should also be considered. In addition, given the importance that extreme storms have on BEBs, with many BEBs typically exhibiting relict post-storm morphology (Costas et al., 2005), future research should focus on storm erosion and recovery processes, including focus on the mechanism by which BEBs recover, as many have an absence of swell waves and yet erosion may not be a one-way process. Despite such research requiring multi-year data sets (e.g. van der Lugt et al., 2023), sediment transport pathways within the estuaries and bays will clarify the relative importance of cross- and long-shore processes and how these relate to estuarine/bay circulation and geomorphology.

We recommend including more focus on mapping and monitoring BEB locations and morphology and long-term monitoring of hydrodynamic processes, drawing inspiration from approaches focused on the open coast (e.g. Luijendijk et al., 2018; Vos et al., 2023). Future studies should consider BEB evolution in relation to evolution and processes of the whole estuary/bay to identify potential mitigation measures based on nature-based solutions. This should include findable, accessible, interoperable and reusable (FAIR) data acquisition programmes that could involve citizen science programmes such as the Victorian Coastal Monitoring Programme (Ierodiaconou et al., 2022) or Coast Snap (Harley and Kinsela, 2022).

The new data sets will be used to develop specific tools to understand and manage BEBs. For example, new quantitative methods for morphodynamic classification that will allow direct comparison of the diverse BEBs and that can be used to underpin management and inform policy. Subsequent research should also focus on developing numerical models to predict BEB evolution at decadal scales. As anthropogenic climate change modifies our environment driving sea-level rise and changes in wave and wind climates, the ecosystem service of coastal protection provided by BEBs, as well as the other contributions to SDGs 11, 14 and 15, will become more important. Understanding BEB morphodynamics is essential for the success of ecosystem restoration practices such as ecosystem restoration or “living shorelines” that are needed to ensure the future of our coastal estuaries and the cities they serve. Long-term coastal prediction can only be meaningful for BEBs if we study their idiosyncrasies and consider them properly in our classification models and coastal management tools and interventions.

## Data Availability

This manuscript does not present any new data.
