# Supplementary material for: Morphodynamics and management challenges for beaches in modified estuaries and bays
Source: Camb Prism Coast Futur. 2024 Aug 27;2:e11. doi: 10.1017/cft.2024.7 (PMC12337578; doi:10.1017/cft.2024.7)
Supplement: Peer review history

# Editor report: Recommendation: Morphodynamics and management challenges for beaches in modified estuaries and bays — R0/PR2

**DOI:** 10.1017/cft.2024.7.pr2

**Affiliations:** Second Institute of Oceanography Ministry of Natural Resources, China

Thank you for submitting your manuscript to our journal. I apologize profusely for the delay in getting this decision to you. I now have received 2 reviews of your manuscript. Both reviewers found this review is important and interesting, which could fill the knowledge gaps in BEBs. However, they also expressed some concerns regarding more case studies and other problems. On the basis of the referees' reports, I recommend ‘Major revision’ and I encourage the authors to revise your manuscript before it could be accepted.

# Editor report: Decision: Morphodynamics and management challenges for beaches in modified estuaries and bays — R0/PR3

**DOI:** 10.1017/cft.2024.7.pr3

**Affiliations:** Geography, Cambridge University, United Kingdom of Great Britain and Northern Ireland

No accompanying comment.

# Editor report: Recommendation: Morphodynamics and management challenges for beaches in modified estuaries and bays — R1/PR5

**DOI:** 10.1017/cft.2024.7.pr5

**Affiliations:** Second Institute of Oceanography Ministry of Natural Resources, China

No accompanying comment.

# Editor report: Decision: Morphodynamics and management challenges for beaches in modified estuaries and bays — R1/PR6

**DOI:** 10.1017/cft.2024.7.pr6

**Affiliations:** Geography, Cambridge University, United Kingdom of Great Britain and Northern Ireland

No accompanying comment.

# Editor report: Recommendation: Morphodynamics and management challenges for beaches in modified estuaries and bays — R2/PR8

**DOI:** 10.1017/cft.2024.7.pr8

**Affiliations:** Second Institute of Oceanography Ministry of Natural Resources, China

No accompanying comment.

# Editor report: Decision: Morphodynamics and management challenges for beaches in modified estuaries and bays — R2/PR9

**DOI:** 10.1017/cft.2024.7.pr9

**Affiliations:** Geography, Cambridge University, United Kingdom of Great Britain and Northern Ireland

No accompanying comment.
